# Alcohol use among adults in Uganda: findings from the countrywide non-communicable diseases risk factor cross-sectional survey

**DOI:** 10.3402/gha.v9.31302

**Published:** 2016-08-03

**Authors:** Steven Ndugwa Kabwama, Sheila Ndyanabangi, Gerald Mutungi, Ronald Wesonga, Silver K. Bahendeka, David Guwatudde

**Affiliations:** 1 Uganda Public Health Fellowship Program, Field Epidemiology Track, Ministry of Health, Kampala, Uganda; 2 Mental Health and Substance Abuse, Ministry of Health, Kampala, Uganda; 3 Control of Non-Communicable Diseases Desk, Ministry of Health, Kampala, Uganda; 4 School of Statistics and Planning, Makerere University College of Business and Management Sciences, Kampala, Uganda; 5 Department of Internal Medicine, St. Francis Hospital Nsambya, Kampala, Uganda; 6 Department of Epidemiology and Biostatistics, School of Public Health, Makerere University College of Health Sciences, Kampala, Uganda

**Keywords:** alcohol use, non-communicable diseases, WHO STEPS methodology, sub-Saharan Africa, Uganda

## Abstract

**Background:**

There are limited data on levels of alcohol use in most sub-Saharan African countries.

**Objective:**

We analyzed data from Uganda's non-communicable diseases risk factor survey conducted in 2014, to identify alcohol use prevalence and associated factors.

**Design:**

The survey used the World Health Organization STEPS tool to collect data, including the history of alcohol use. Alcohol users were categorized into low-, medium-, and high-end users. Participants were also classified as having an alcohol-use-related disorder if, over the past 12 months, they were unable to stop drinking alcohol once they had started drinking, and/or failed to do what was normally expected of them because of drinking alcohol, and/or needed an alcoholic drink first in the morning to get going after a heavy drinking session the night before. Weighted logistic regression analysis was used to identify factors associated with medium- to high-end alcohol use.

**Results:**

Of the 3,956 participants, 1,062 (26.8%) were current alcohol users, including 314 (7.9%) low-end, 246 (6.2%) medium-end, and 502 (12.7%) high-end users. A total of 386 (9.8%) were classified as having an alcohol-use-related disorder. Male participants were more likely to be medium- to high-end alcohol users compared to females; adjusted odds ratio (AOR)=2.34 [95% confidence interval (CI)=1.88–2.91]. Compared to residents in eastern Uganda, participants in central and western Uganda were more likely to be medium- to high-end users; AOR=1.47 (95% CI=1.01–2.12) and AOR=1.89 (95% CI=1.31–2.72), respectively. Participants aged 30–49 years and those aged 50–69 years were more likely to be medium- to high-end alcohol users, compared to those aged 18–29 years, AOR=1.49 (95% CI=1.16–1.91) and AOR=2.08 (95% CI=1.52–2.84), respectively.

**Conclusions:**

The level of alcohol use among adults in Uganda is high, and 9.8% of the adult population has an alcohol-use-related disorder.

## Introduction

According to estimates by the World Health Organization (WHO), there are 2 billion people worldwide who consume alcoholic beverages (1). When consumed in moderate amounts (up to one standard drink per day for women, and one to two standard drinks per day for men), alcohol consumption has been found to be associated with decreased risk of overall mortality and a number of chronic non-communicable diseases (NCDs), including coronary artery disease, diabetes mellitus, congestive heart failure, and stroke (2–9). However, when taken in large amounts, the benefits that accrue from moderate alcohol use are negated, leading to elevated risks of other NCDs like cancers, injuries, and a wide range of social problems (1, 10–12). Although alcohol consumption is largely socially acceptable in many societies (13, 14), it has substantial effects on the health and well-being of individuals and the community as a whole. Of the estimated 2 billion people who consume alcohol globally, almost 80 million have diagnosable alcohol-use disorders (1). In the year 2000, 3.2% of all deaths globally could be attributed to alcohol consumption (15).

Uganda has previously been reported to have one of highest levels of alcohol consumption in the East African region, with an annual per capita alcohol consumption of 23.7 liters (16). Some studies have also associated alcohol use with an increased risk of road traffic accidents (17), HIV infection (18), risky sexual behaviors (19), sexual coercion (20), and intimate partner violence (21). However, most of these studies were conducted on limited population groups in particular geographic locations using differing methodologies and are therefore not sufficient for clarifying the national prevalence of alcohol use. In 2014, a countrywide NCDs risk factor survey was conducted in Uganda to provide baseline estimates of the prevalence of common risk factors for NCDs in the country. Between May and August 2015, we analyzed data from this survey to estimate the prevalence of alcohol use and its distribution by geographical region, determine the prevalence of problem drinking, and identify associated factors in the Ugandan population.

## Methods

A cross-sectional study design was used to conduct the NCDs risk factor survey, between April and July 2014. The survey used the WHO STEP-wise approach, which is a standardized method of analyzing risk factors for NCDs (22). A detailed description of the methods use in the Uganda survey is reported elsewhere (23, 24). Here, we only describe methods relevant to results presented in this article.

### Measurements

Measurements followed the three steps of the WHO STEPS methodology (22). STEP 1 comprised of administration of a questionnaire to obtain information on the demographic, socio-economic, and behavioral characteristics of participants. In STEP 2, we made physical measurements. In STEP 3, we made biochemical measurements. Alcohol use was measured as part of STEP 1. Participants were asked whether they had ever consumed any alcoholic beverage or not. Those reporting that they had ever consumed alcohol were asked to provide details of alcohol consumption over the past 12 months and over the past 30 days. Details included the type of alcohol consumed, the frequency of consumption, and the average quantity consumed per sitting. Based on the information provided, the interviewer identified the type of alcohol and recorded the type of alcohol consumed as a ‘beer’, a ‘wine’, or a ‘spirit’ (whisky or gin). Further, interviewers quantified the amount consumed in terms of the numbers of standard drinks. WHO defines one standard alcoholic drink as any alcohol drink that contains 10 g of pure alcohol (25). The following measures were taken as equivalent to one standard alcoholic drink: (1) a 285-ml bottle or can of beer, (2) a 120-ml glass of wine (factory distilled or locally brewed), and (3) a 30-ml glass/tot of a spirit or gin (factory distilled or locally brewed) (26). Showcards with local pictures of the different types of alcoholic beverages commonly consumed in Uganda were used to help participants identify the types of alcoholic beverages they may have consumed. The pictures on these showcards had previously been adapted by the investigators and used during the training of the interviewers.

### Ethics

Written informed consent was obtained from the consenting sampled subjects before conducting any study procedures. The conduct of the survey was approved by the Institutional Review Committee of Nsambya Hospital, Kampala, Uganda, and registered by the Uganda National Council for Science and Technology.

### Statistical analysis

We grouped participants into three broad categories of alcohol use comprising (1) ‘never users’ that included participants who had never consumed alcohol, (2) ‘ever used but were currently not using’ that included participants who had ever consumed alcohol but had not done so within the past 30 days, and (3) ‘current users’ that included participants reporting that they had consumed alcohol within the 30 days preceding the survey.

Current alcohol users were further categorized into high-end, medium-end, and low-end alcohol users. High-end users were participants reporting binge drinking in the past 30 days (an equivalent of more than six standard drinks of alcohol in one sitting), and/or consuming an equivalent of more than six standard drinks of alcohol on average per occasion among men or an equivalent of more than four standard drinks of alcohol on average per occasion among women. Medium-end alcohol users were participants reporting consuming an equivalent of four to six standard drinks of alcohol on average per occasion among men or two to four standard drinks of alcohol on average per occasion among women. Low-end alcohol users were participants who reported consuming an equivalent of fewer than four standard drinks of alcohol on average per occasion for men and for women fewer than two standard drinks of alcohol on average per occasion (26).

Participants were classified as having an alcohol drinking disorder if, over the 12 months preceding the interview, at least once a month they were unable to stop drinking alcohol once they had started drinking, and/or failed to do what was normally expected of them because of drinking alcohol, and/or needed an alcoholic drink first in the morning to get going after a heavy drinking session.

We report the prevalence of alcohol use using the above categories. Pearson's chi-square test statistic was used to evaluate differences in the distribution of alcohol use by categories of participant characteristics. Because large quantities of alcohol use have been associated with higher risk for a number of health conditions (11), we further categorized alcohol use into two, that is, medium- and high-end alcohol use versus low-end and non-current users.

To identify factors associated with medium- to high-end alcohol use, a weighted logistic regression analysis was used to estimate both crude and adjusted odds ratios (AORs) and their corresponding 95% confidence intervals (CIs). Sampling selection weights were used. All statistical analyses were performed using STATA version 12.

## Results

### Characteristics of participants

Of the 3,987 subjects that participated in the NCDs risk factor survey, 3,956 provided information on their alcohol consumption history and are included this analysis. Of the 3,956 participants, 2,371 (59.9%) were females, 3,274 (82.7%) were aged 18–49 years, 2,875 (72.7%) lived in rural areas, and only 654 (9.5%) reported that they had not attended any schooling. There were 2,534 (64.1%) participants who were currently married and 2,585 (65.4%) had some form of employment. The average age of participants was 34.9 (standard deviation=13.3). A summary of selected characteristics of the participants is presented in Table 1.

**Table 1:** Characteristics of participants

Characteristics	*n*	Summary measure (%)
Region of residence		
Eastern		24.2
Central	1,290	32.6
Northern	768	19.4
Western	941	23.8
Urban–rural residence		
Urban	1,081	27.3
Rural	2,875	72.7
Sex		
Female	2,371	59.8
Male	1,585	40.2
Age		
18–29	1,608	40.6
30–49	1,666	42.1
50–69	682	17.3
Mean±SD	3,956	34.9±13.3
Highest level of education completed		
No formal schooling	644	16.3
Primary school	1,615	41.0
Secondary school	1,309	33.2
University or higher	374	9.5
Ethnicity		
Basoga	316	8.0
Banyankole	446	11.3
Baganda	775	19.6
Banyoro/Batoro	257	6.5
Bakiga	253	6.4
Lugbara/Madi	247	6.2
Other	1,661	42.0
Marital status		
Never married	625	15.8
Currently married	2,534	64.1
Cohabiting	92	2.3
Other	703	17.8
Employment over the past 12 months		
Unemployed	1,370	34.6
Employed	2,585	65.4

### Prevalence and distribution of alcohol use

**Fig. 1:**
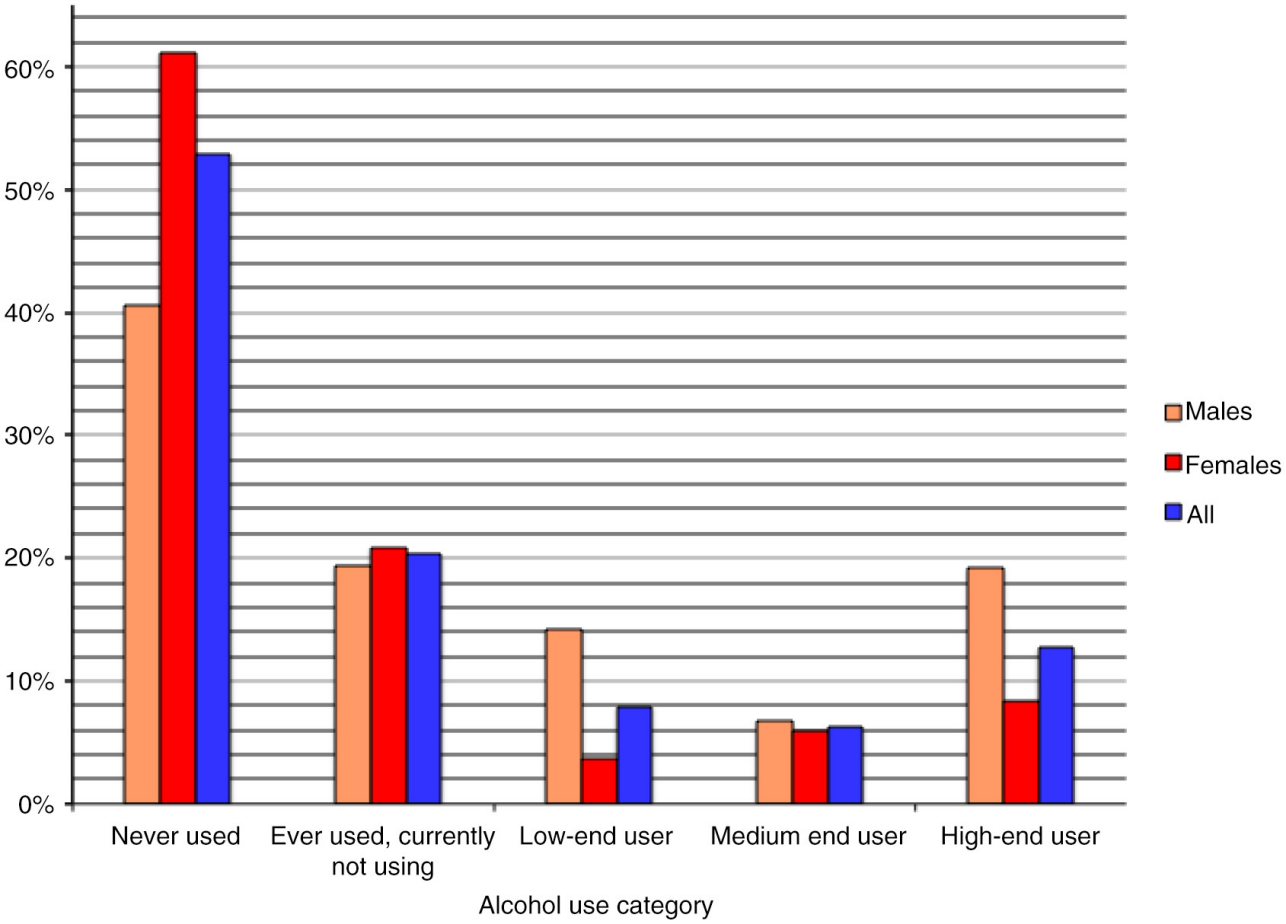
Distribution of alcohol use history by the gender of the participants.

Of the 3,956 participants, 1,062 (26.8%) reported that they were current users of alcohol comprising 314 (7.9%) low-end users, 246 (6.2%) medium-end users, and 502 (12.7%) high-end users. A total of 802 (20.3%) participants reported that they had ever consumed alcohol but were currently not using alcohol, and 2,092 (52.9%) reported that they had never consumed any alcoholic drink (Fig. 1). A total of 386 (9.8%) participants were classified as having an alcohol-use-related disorder.

The prevalence of medium- to high-end alcohol use was highest among residents in the northern region of the country at 23.2%, followed by residents in the western region at 21.4%, the central region at 18.5%, and the eastern region at 13.7% (unadjusted *p*<0.001). There was no significant difference in the prevalence of medium- to high-end alcohol use between urban and rural residents (unadjusted *p*=0.077). The prevalence of medium- to high-end alcohol use was higher among males at 25.9% compared to females at 14.3% (unadjusted *p*<0.001). Prevalence increased with age, being 13.2% among those aged 18–29 years, and 25.4% in those aged 50–69 years (unadjusted *p*<0.001). The distribution of medium- to high-end alcohol use by other participant characteristics is summarized in Table 2.

**Table 2:** Crude and adjusted odds ratios (ORs) of being a medium- or high-end alcohol user compared to a never, stopped, or low-end alcohol user

Variable	*n*	Number of medium- to high-end users (%)	Crude OR (95% CI)	Adjusted OR (95% CI)^a^
Urban–rural residence				
Urban	1,081	185 (17.1)	1.0	1.0
Rural	2,875	563 (19.6)	1.16 (0.86–1.54)	1.18 (0.91–1.52)
Region of residence				
Eastern	957	131 (13.7)	1.0	1.0
Central	1,290	238 (18.5)	1.27 (0.85–1.88)	1.47 (1.01–2.12)
Northern	768	178 (23.2)	2.12 (1.41–3.20)	1.89 (1.31–2.72)
Western	941	201 (21.4)	1.13 (0.70–1.86)	1.15 (0.74–1.79)
Sex				
Females	2,371	338 (14.3)	1.0	1.0
Males	1,585	410 (25.9)	2.58 (2.00–3.33)	2.34 (1.88–2.91)
Age				
18–29	1,608	212 (13.2)	1.0	1.0
30–49	1,666	363 (21.2)	1.40 (1.06–1.85)	1.49 (1.16–1.91)
50–69	682	173 (25.4)	1.59 (1.11–2.26)	2.08 (1.52–2.84)
Ethnicity				
Basoga	316	20 (6.3)	1.0	1.0
Banyankole	446	94 (21.1)	4.77 (2.19–10.36)	5.21 (2.55–10.6)
Baganda	775	123 (15.9)	2.42 (1.20–4.87)	2.29 (1.20–4.38)
Banyoro/Batoro	257	58 (22.6)	4.14 (1.87–9.18)	4.47 (2.16–9.22)
Bakiga	253	66 (26.1)	5.40 (2.45–11.92)	6.30 (3.01–13.1)
Lugbara/Madi	247	38 (15.4)	1.42 (0.62–3.24)	1.76 (0.82–3.77)
Other	1,661	348 (21.0)	3.51 (1.92–6.50)	3.92 (2.22–6.91)
Employment				
Unemployed	2,585	531 (20.5)	1.0	1.0
Employed	1,370	217 (15.8)	1.10 (0.83–1.45)	0.98 (0.76–1.26)
Level of education				
No formal schooling	664	147 (22.8)	1.0	1.0
Primary	1,615	311 (19.3)	0.86 (0.62–1.18)	0.85 (0.64–1.14)
Secondary	1,309	207 (15.8)	0.70 (0.48–1.02)	0.72 (0.51–1.02)
University or higher	373	78 (20.9)	0.68 (0.40–1.17)	0.72 (0.46–1.13)
Marital status				
Never married	625	88 (14.1)	1.0	1.0
Currently married	2,534	489 (19.3)	1.03 (0.68–1.55)	1.07 (0.74–1.55)
Cohabiting	92	12 (13.0)	1.04 (0.44–2.47)	0.98 (0.44–2.19)
Other	703	159 (222.6)	1.32 (0.80–2.20)	1.39 (0.90–2.16)
Hypertensive				
No	2,942	512 (17.4)	1.0	1.0
Yes	939	217 (23.1)	1.24 (0.96–1.60)	1.16 (0.92–1.48)
FPG (mmol/L)^b^				
<6.1	3,538	666 (18.8)	1.0	1.0
6.1–6.9	82	22 (26.8)	1.77 (0.91–3.42)	1.56 (0.83–2.92)
≥7.0, or on DM Rx	42	11 (23.9)	2.09 (0.78–5.62)	1.71 (0.68–4.32)

a Adjusted for sex, age, region of residence, and ethnicity.

b FPG=fasting plasma glucose in millimoles per liter (mmol/L).

### Factors associated with medium to high-end alcohol use

Factors found to be significantly associated with medium- to high-end alcohol use were sex, age, region of residence, and ethnicity. Male participants were more likely to be medium- or high-end alcohol users compared to females with an AOR of 2.34 [95% CI=1.88–2.91]. Participants aged 30–49 years were more likely to be medium- or high-end alcohol users compared to those aged 18–29 years with an AOR of 1.49 (1.16–1.91), and so were participants aged 50–69 years with an AOR of 2.08 (1.52–2.84).

Compared to participants in the eastern region of Uganda, participants in the central region were more likely to be medium- or high-end alcohol users with an AOR of 1.47 (1.01–2.12), and so were participants in northern Uganda with an AOR of 1.89 (1.31–2.72). Compared to the Basoga ethnic group, participants of Banyankole ethnic group were significantly more likely to be medium- or high-end alcohol users with an AOR of 5.21 (2.55–10.6) as were participants of Baganda ethnic group with an AOR of 2.29 (1.20–4.38) and participants of Banyoro/Batoro ethnic group with an AOR of 4.47 (2.16–9.22). Also, compared to the Basoga ethnic group, participants of the Bakiga ethnic group were more likely to be medium- or high-end alcohol users with an AOR of 6.30 (3.01–13.1) as were participants of Lugbara/Madi ethnic group with an AOR of 1.76 (0.82–3.77) and participants of other ethnic groups with an AOR of 3.92 (2.22–6.91) (Table 2).

Factors that did not attain statistical significance were employment status (*p*=0.498), level of education (*p*=0.310), being hypertensive (*p*=0.106), body mass index (*p*=0.301), fasting plasma glucose status (*p*=0.090), being an urban–rural resident (*p*=0.330), and marital status (*p*=0.433).

## Discussion

This is the first survey in Uganda to provide a countrywide estimate of the prevalence of alcohol use. Findings show an overall prevalence of current alcohol use of 26.8%, with nearly 10% of the population having an alcohol use disorder. All previous studies in Uganda on alcohol use have been conducted in small localities or subpopulations. The Gender Alcohol and Culture International (GENACIS) study found that 40.1% of males and 20.3% of females were high-end users of alcohol (27), in contrast with 25.9% of men and 14.3% of women in our survey. However, data for the GENACIS study were collected from only four out of the 110 districts of the country. Other smaller scope studies include one study among people receiving HIV counseling and testing that reported a prevalence of alcohol use in this group of 30% (28), two studies among HIV-infected patients in southwestern Uganda, of which one gave an estimate of 21% (29) and another gave 13% (30), and another study among rural residents in southwestern Uganda at 8% (31).

We also found a high prevalence of medium- to high-end alcohol use at 18.9%. The most recent Global Status Report on Alcohol and Health of 2014 gave the average annual per capita alcohol consumption in Uganda to be 23.7 liters, with 3.4% of the population being heavy episodic drinkers (drinking six or more standard drinks of alcohol in the past 30 days) (16). This is equivalent to approximately 6.4 standard drinks of alcohol per day per person. Indeed, 9.8% of participants in our survey had an alcohol-use-related disorder. It, therefore, appears that, among alcohol users, some are heavy consumers, a factor that increases overall per capita alcohol consumption.

Other countries in the sub-Saharan Africa region that have also found similar levels of current alcohol use include Tanzania 29.3% (32), while higher levels have been shown in Zimbabwe 58% (33) and Ethiopia 45.7% (34). Lower levels have been shown in Botswana 18.7% (35) and Zambia 20.8% (36). However, findings from our survey are in stark contrast with previous reports in the local media and other reports that have ranked Ugandans as being the highest consumers of alcohol in sub-Saharan Africa (37, 38).

We found a significant difference in the prevalence of medium- to high-end alcohol consumption among men compared to women. Similar findings have been made by other researchers (13, 39–41). In Uganda, this difference might be attributed to culture and gender-based distinctions between the roles, responsibilities, and expectations of men and women. Qualitative investigations into the social norms related to alcohol-use behavior among men and women in Uganda have shown that alcohol use among men is associated with masculinity (42), social independence, and freedom from domestic responsibilities (43). Among girls, the use of alcohol is associated with a lack of respect (42) and a defiance of the feminine ideals of domesticity (43), which can attract social sanctions (14). These beliefs may be responsible for the lower prevalence of alcohol use among women. Other countries in the sub-Saharan region that have conducted the WHO STEPS survey, including the United Republic of Tanzania, Zambia, Ethiopia, Botswana, and Zimbabwe, found higher levels of alcohol use among men compared with women.

We also found that older participants were more likely to be medium- to high-end alcohol users than younger participants. This could be attributed to a culture that socially embraces alcohol use (42), as well as stress factors, such as deterioration in health, the experience of traumatic events (44), and high levels of unemployment (27, 45) among older people. Some researchers have noted that in countries with a heavy HIV/AIDS burden, this can be a stress factor associated with alcohol use (27, 46), while others have noted that alcohol use may in fact be lower among diagnosed HIV-infected adults because of the advice they receive from clinicians, threats to financial security, and the negative impact on social standing (47). Also, older people are more likely to be part of social events, such as betrothals, weddings, and burials, most of which involve alcohol use as a part of the cultural celebration (43).

The survey also showed a higher prevalence of alcohol use among residents in the northern region compared to the eastern region of Uganda. It is likely that the traumatic stress, resulting from over three decades of civil war, cattle raiding, and armed banditry in the northern region, has led residents to consume alcohol as a coping mechanism (44, 48). The high level of alcohol use in northern Uganda is similar to findings from the Uganda Demographic and Health Survey (2001), in which the northern region had the highest percentage of current alcohol users (49).

### Strengths and limitations

The results of the survey should be interpreted in light of certain limitations. The fact that the study was based on a cross-sectional study design precludes the inference of a causal association between any of the factors examined and alcohol use. However, the sampling procedure used was robust enough for the findings to be generalizable to the Ugandan population. Also, the use of the standardized WHO STEPS tool in the assessment of alcohol use means that findings from the survey can be compared with those from other countries that have used a similar methodology.

## Conclusions

Although comparatively lower than in other countries in the sub-Saharan region, the prevalence of alcohol use in Uganda is still high, with an estimated 10% of the population having an alcohol-use-related disorder. Males, older individuals, residents in northern, western, and central Uganda, and individuals of particular ethnic backgrounds are more likely to be medium- to high-end alcohol users. Public health interventions aimed at reducing the levels of alcohol use in the country need to take these population characteristics into consideration.

## Authors' contributions

GM, SKB, and DG conceived and designed the study. SKB, DG, and GM implemented the study. SNK and DG analyzed the data. DG and SNK had primary responsibility for final content. All authors participated in the writing of this article, and read and approved the final manuscript.

## Paper context

Uganda has previously been reported to have the highest levels of alcohol consumption in the East African region. These data were based on studies conducted on limited population groups, and their findings therefore do not sufficiently clarify the extent of alcohol use in the country. Alcohol use data reported in this article are based on a countrywide sample that provides representative benchmark data for informing policy on the control and regulation of alcohol use in Uganda.
